# Natural Flavonoid Pectolinarigenin Alleviated Hyperuricemic Nephropathy via Suppressing TGFβ/SMAD3 and JAK2/STAT3 Signaling Pathways

**DOI:** 10.3389/fphar.2021.792139

**Published:** 2022-01-27

**Authors:** Qian Ren, Bo Wang, Fan Guo, Rongshuang Huang, Zhouke Tan, Liang Ma, Ping Fu

**Affiliations:** ^1^ Kidney Research Institute, National Clinical Research Center for Geriatrics and Division of Nephrology, West China Hospital of Sichuan University, Chengdu, China; ^2^ Division of Nephrology, ZunYi Medical University Affiliated Hospital, ZunYi, China

**Keywords:** hyperuricemic nephropathy, pectolinarigenin, renal fibrosis, inflammation, fatty acid-binding protein 4

## Abstract

Natural flavonoid pectolinarigenin (PEC) was reported to alleviate tubulointerstitial fibrosis of unilateral ureteral obstruction (UUO) mice in our previous study. To further investigate nephroprotective effects of PEC in hyperuricemic nephropathy (HN), adenine and potassium oxonate induced HN mice and uric acid-treated mouse kidney epithelial (TCMK-1) cells were employed in the study. As a result, PEC significantly lowered serum uric acid level and restored hyperuricemia-related kidney injury in HN mice. Meanwhile, PEC alleviated inflammation, fibrosis, and reduced adipokine FABP4 content in the kidneys of HN mice and uric acid-treated TCMK-1 cells. Mechanistically, PEC inhibited the TGF-β1 expression as well as the phosphorylation of transcription factor SMAD3 and STAT3 to regulate the corresponding inflammatory and fibrotic gene expression in kidney tissues. In conclusion, our results suggested that PEC could inhibit the activation of SMAD3 and STAT3 signaling to suppress inflammation and fibrosis, and thereby alleviate HN in mice.

## Introduction

Hyperuricemia (HUA) is a metabolic disease characterized by elevated uric acid (UA) in blood, the prevalence of which has increased worldwide substantially in recent years (Dehlin et al., 2020; Dalbeth et al., 2021). Studies showed that HUA was highly associated with diabetes, hypertension, cardiovascular diseases, and chronic kidney diseases (CKD) (Pascart and Lioté, 2019). As serum UA is mainly secreted by the renal proximal tubules, HUA is a frequent finding in person with CKD due to decreased UA clearance (Johnson et al., 2013). In return, recent evidence suggested that HUA independently predicted the development and progression of CKD (Landa, 2018; Balakumar et al., 2020).

HUA-induced kidney injury, known as hyperuricemic nephropathy (HN), is featured by urate deposition, arteriolosclerosis, glomerular hypertension, and tubulointerstitial fibrosis and would eventually progress into end-stage renal diseases (ESRD) (Liu et al., 2015). The mechanism of HN is complex with many factors such as crystalline effect, oxidative stress, rennin-angiotensin system activation, and tubular epithelial cell transition having been postulated. Though controversial, accumulating data suggested that the UA-lowering treatments could slower the progression of CKD (Liu et al., 2021; Yanai et al., 2021). Current first-line urate-lowering drugs are mainly xanthine oxidase (XO) inhibitors and uricosuric agents, both of which have limited application in clinics because of their low selectivity or toxic reaction (Balakumar et al., 2020). Hence, it is imperative to develop a new therapeutic agent for HN.

Flavonoid pectolinarigenin (PEC), a plant secondary metabolite that has various biological effects, is one of the major compounds in *Cirsium setidens* (Lee et al., 2017). Studies reported that pectolinarigenin conducted antimicrobial, antioxidant, anti-inflammatory, and antidiabetic activities (Cheriet et al., 2020). Meanwhile, PEC was found to suppress lipopolysaccharide-induced inflammation via NF-κB and MAPK pathways (Heimfarth et al., 2021). In addition, PEC derivatives exhibited selective activity against tumor cells, exhibiting anti-carcinogenic activity (Deng et al., 2020). In our previous study, PEC treatment exerted an anti-fibrotic effect in a mouse model of unilateral ureteral obstruction (UUO). However, the effect of PEC on HN remains unknown. The current study aimed to evaluate whether PEC could be a candidate for HN treatment and explore possible mechanisms.

## Materials and Methods

### Chemicals and Materials

Pectolinarigenin (PEC) was obtained from Chengdu Chroma-Biotechnology Co., Ltd. (purity ≥99.0%). Antibodies against GAPDH, α-tubulin, fatty acid-binding protein (FABP4), IL-6, alpha-smooth muscle actin (α-SMA), janus kinase 2 (JAK2), p-JAK2, Smad3 and p-Smad3, and cleaved caspases 3 (C casp 3) were purchased from Hangzhou HuaAn Biotechnology Co., Ltd. (Hangzhou, China). Antibodies against Collagen-1(Col I), Fibronectin (FN), signal transducer and activator of transcription 3 (STAT3), p-STAT3, BAX, and Bcl2 were bought from Abcam (Cambridge, MA, United States). Anti-TNF-α antibody was bought from Affinity Bioscience (Cincinnati, OH, United States).

### Animals

The HN model was established in male C57BL/6J mice (8–10 weeks old; 20–25 g) provided by the Animal Laboratory Center of Sichuan University (Chengdu, China). Forty mice were randomly assigned to five groups: Control (*n* = 8), HN (*n* = 8), Allopurinol (*n* = 8), PEC 25 mg/kg (*n* = 8), PEC 50 mg/kg (*n* = 8). The HN model was established by feeding mice with a mixture of adenine (0.16 g/kg) and potassium oxonate (2.4 g/kg) every other day for 4 weeks, as previously described (Ren et al., 2021). Allopurinol (10 mg/kg) and PEC (25 and 50 mg/kg) were orally given daily during the experiment along with HN establishment (for 4 weeks). The mice were sacrificed, and the kidneys were collected at the end of study. Ethical approval was granted by the Animal Ethics Committee of West China Hospital of Sichuan University (No. 2020061A).

### Histologic Examination

Tissue sections were fixed with 10% phosphate buffered formalin and embedded in paraffin after dehydrating. Kidney slides of 4-μm thickness were subject to PAS staining for morphologic analysis and Masson staining for fibrotic analysis (Ren et al., 2021). Six pictures (×400) per kidney were randomly captured by light microscopy for semiquantitative analysis. The tubular injury score was evaluated on the base of histopathology of injured/damaged renal tubules and was graded from 0 to 4 (0: 0%; 1: <25%; 2: 26–50%; 3: 51–75%; 4: ≥76% of injured/damaged renal tubules) (Liu et al., 2015). The collagen positive area was measured by the ImageJ software.

### Western Blotting Analysis

Total proteins were isolated from frozen kidney tissue or mouse kidney epithelial cells (TCMK-1, ATCC^®^ CCL-139™, Beijing bnbio Co., Ltd., Beijing, China) using radio immune precipitation (RIPA) lysis buffer (P0013B, Beyotime Biotechnology, China) and quantified using a Pierce™ BCA Protein Assay Kit (23225, Thermo Scientific, Billerica, MA, United States). Equal amounts of protein lysate were separated on 10–12% SDS-PAGE as previously described (Ren et al., 2021). Immunoblots were visualized by the Immobilon Western Chemiluminescent HRP Substrate (WBKLS0500, Millipore Corporation, Billerica, MA, United States) with Bio-Rad Chemi Doc MP and densitometered by ImageJ 6.0 software (National Institutes of Health, Bethesda, MD, United States).

### Immunohistochemistry Staining

Immunohistochemical staining was performed as previously described (Ren et al., 2021). The following primary antibodies were used: anti-α-SMA (1:100, Huabio), anti-STAT3 (1:200, Abcam), anti-p-STAT3 (1:100, Abcam), anti-FABP4 (1:100, Huabio). Images were examined and acquired with an AxioCamHRc digital camera (Carl Zeiss, Jena, Germany).

### Quantitative Real-Time PCR Analysis

Total RNA in kidney tissues of mice or TCMK-1 cells was isolated with a total RNA extraction Kit (TP-01121, Foregene, Chengdu, China) according to the manufacturer’s instructions. The concentration of mRNA was determined by a Scan Drop 100 (Analytik Jena, Thuringia, Germany) determiner. Quantitative real-time PCR assays were performed on a PCR system (CFX Connect; Bio-Rad, Hercules, CA, United States). The sequences of primers are shown in Supplementary Table S1. Statistical analysis was conducted using the comparative 2^−ΔΔCT^ method with GAPDH or *β-actin* as the internal standard.

### RNA-Seq Transcriptomic Assay

Total RNA was extracted from kidney tissues with Trizol reagent (Invitrogen, Carlsbad, CA, United States). Total RNA quality was assessed using the RNA 6000 Nano LabChip Kit (Agilent, CA, United States) of the Agilent Bioanalyzer 2100 system. The RNA-seq were performed by LC-BIO Bio-tech Ltd. (Hangzhou, China). Differentially expressed genes were defined as those with fold changes ≥1.5 and *p* ≤ 0.05. Gene Ontology (GO) functions and Kyoto Encyclopedia of Genes and Genomes (KEGG) pathway enrichment analysis were performed using the OmicStudio tools at https://www.omicstudio.cn/tool.

### Cell Culture and Treatment

TCMK-1 cells were cultured in DMEM (Sigma-Aldrich) supplemented with 5% FBS (SH30084.03, Hyclone, Australia) in a humidified atmosphere (5% CO_2_, 37°C). After incubating with DMEM containing 0.5% FBS for 24 h, cells were exposed to UA (800 μM) and treated with PEC of various concentrations (25, 50, 100, 150, and 300 μM) for 24 h.

### Cell Viability Assay

A Cell Counting Kit-8 assay (CCK-8, Meilunbio, Dalian, China) was employed to assess cytotoxicity. Briefly, TCMK-1 cells were seeded into a 96-well plate at a density of 5,000–10,000 cells/well and incubated with various concentrations of PEC (25, 50, 100, 150, and 300 μM) with or without UA. Cells cultured in DMEM containing the same amount of DMSO were used as control. Twenty-four hours after incubation, the cells were incubated with 10% CCK-8 reagent for 1 hour (37°C, dark). Finally, the absorbance was detected by a microplate reader (Synergy Mx, Biotek, Winooski, VT, United States) at a wavelength of 450 nm.

### Statistical Analysis

Results are presented as the mean ± SD. Differences among multiple groups were compared using one-way analysis of variance (ANOVA) and a Tukey-Kramer *post hoc* test. Comparisons between two groups were performed using the two-tailed *t* test. All statistics were performed using Prism software (ver. 6.01; GraphPad, San Diego, CA, United States) and *p* < 0.05 was considered statistically significant.

## Results

### Pectolinarigenin Lowered Serum Uric Acid Level, Improved Kidney Function, and Attenuated Renal Morphology in Hyperuricemic Nephropathy Mice

**FIGURE 1:**
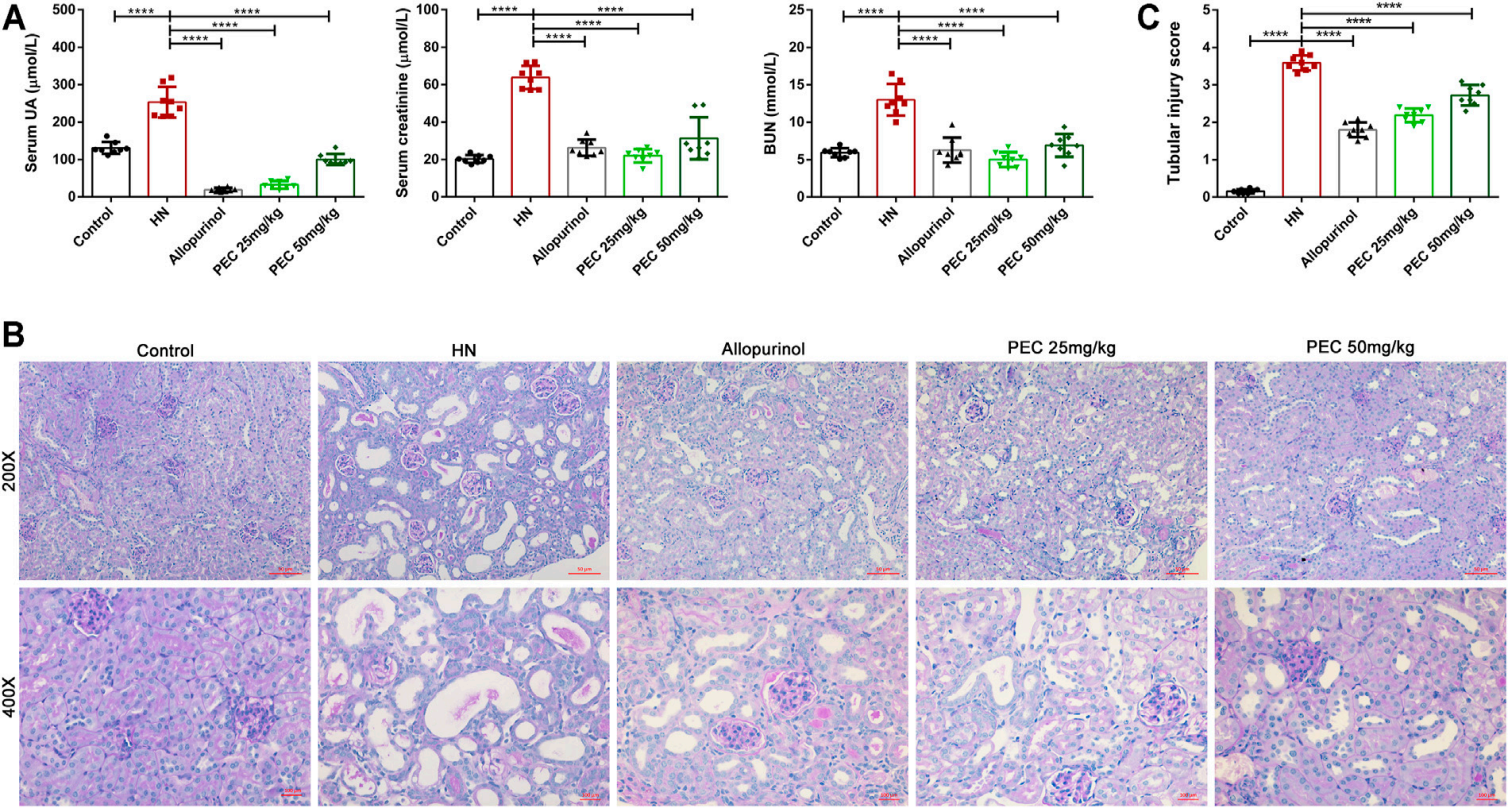
Effects of PEC on serum UA and kidney function in HN mice. **(A)** Biochemical parameters (Serum UA, Serum creatinine, BUN) in mice. **(B)** PAS staining in the kidney. **(C)** Kidney injury score (*n* = 8). All data are represented as the mean ± SD. **p < 0.01, ***p < 0.001, ****p < 0.0001.

Administration of adenine and potassium oxonate successfully induced HN experimental mice as evidenced by increased serum UA level and aggravated kidney function. According to Figure 1A, the serum levels of UA (253.4 ± 14.49 μM vs. 131.0 ± 5.631 μM, *p* < 0.05), blood urea nitrogen (BUN) (13.00 ± 0.7513 mM vs. 5.939 ± 0.2137 mM, *p* < 0.05), and creatinine (63.86 ± 2.183 μM vs. 20.33 ± 0.7468 μM, *p* < 0.05) were significantly higher than those of control mice. After allopurinol and PEC treatment, the serum levels of UA, urea nitrogen, and creatinine were significantly decreased, and PEC at a dose of 25 mg/kg seems more superior in reducing above indexes than PEC with a higher dose (50 mg/kg). Observation of kidney changes in mice by PAS staining also showed that pathological changes in HN mice were alleviated by allopurinol and PEC treatment (Figure 1B). However, tubular injury scores of mice in the PEC 25 mg/kg group were similar to those of the PEC 50 mg/kg group, indicating no superiority of low dose PEC in attenuating renal histopathology (Figure 1C).

### Analysis of Renal Transcriptome in Hyperuricemic Nephropathy Mice

**FIGURE 2:**
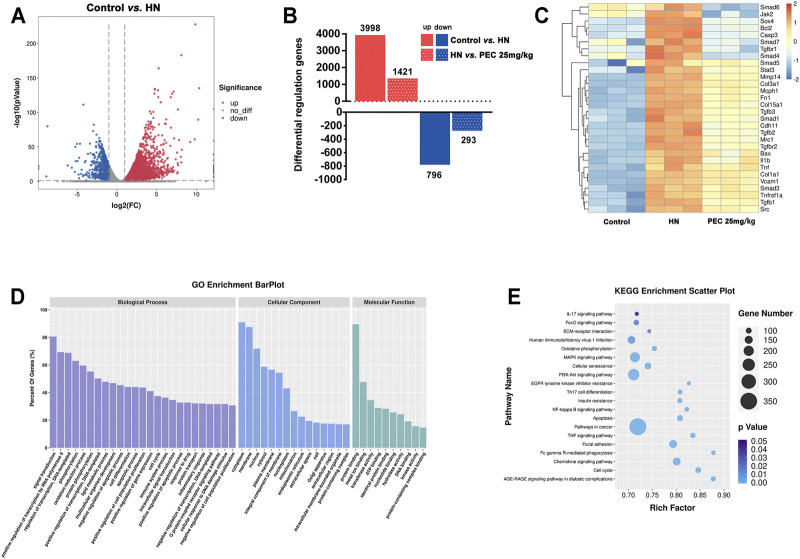
Effects of PEC on gene expression in kidneys of HN mice. **(A)** Volcano plot of gene expression difference between Control and HN groups. **(B)** Differentially regulated genes difference between Control, HN, and PEC 25 mg/kg. **(C)** Heatmap of gene expression difference among Control, HN, and PEC 25 mg/kg. **(D)** GO analysis of differentially expressed genes. **(E)** KEGG analysis of differentially expressed genes.

To reveal the mechanism by which PEC improved kidney injury in HN mice, the RNA-seq analysis was applied. The results of volcano plot showed significantly different gene expression profile between control and HN mice (Figure 2A). Among these differentially expressed genes, 796 genes were up-regulated and 1,998 genes were down-regulated in kidneys of HN mice in comparison with control mice (*p* < 0.05). Remarkably, PEC 25 mg/kg significantly reversed the change of 1,421 down-regulated and 293 up-regulated genes (*p* < 0.05) (Figure 2B). The significant PEC-modulated genes were illustrated by heatmap in Figure 2C, and genes related to apoptosis (Bax), inflammation (il1b, Tnf), and fibrosis (Col-1a1, Fn1) were seen. Further GO and KEGG analysis also suggested that these differentially expressed genes were involved in processes of lipid metabolism, apoptosis, inflammatory response, and fibrogenesis (Figures 2D,E).

### Pectolinarigenin Ameliorated Apoptosis, Reduced Expression of Proinflammatory Genes, and Improved Fibrosis in Kidneys of Hyperuricemic Nephropathy Mice

**FIGURE 3:**
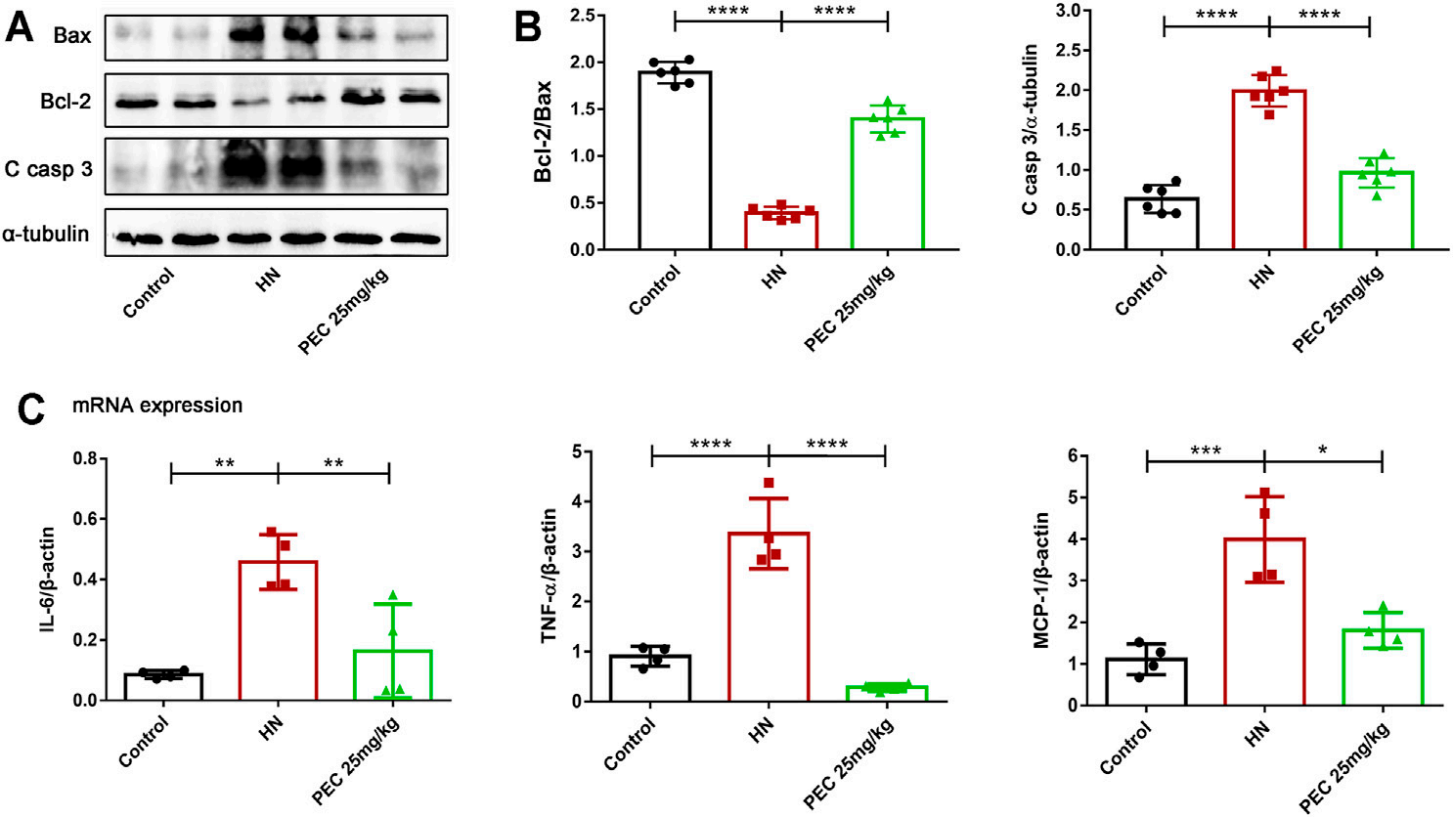
Effects of PEC on kidney apoptosis and inflammation in HN mice. **(A,B)** The Bcl2/Bax, Cleaved caspase 3 (C casp 3) protein levels normalized by β-actin. **(C)** The mRNA expressions of IL-6, TNFα, and MCP-1 measured by real-time PCR analysis. All data are represented as the mean ± SD. *p < 0.05, **p < 0.01, ****p < 0.0001.

**FIGURE 4:**
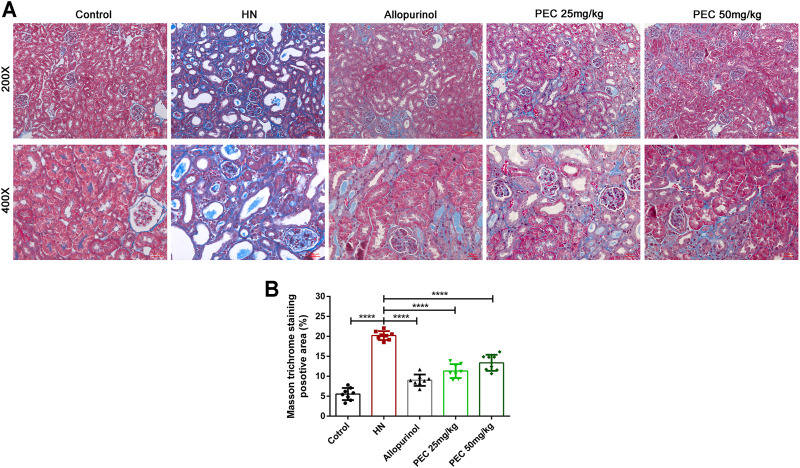
Effects of PEC on kidney fibrosis in HN mice. **(A)** Masson’s trichrome staining of mouse kidneys (×200 and ×400). **(B)** Quantitation of positive area in Masson’s trichrome staining (*n* = 8). All data are represented as the mean ± SD. ****p < 0.0001.

**FIGURE 5:**
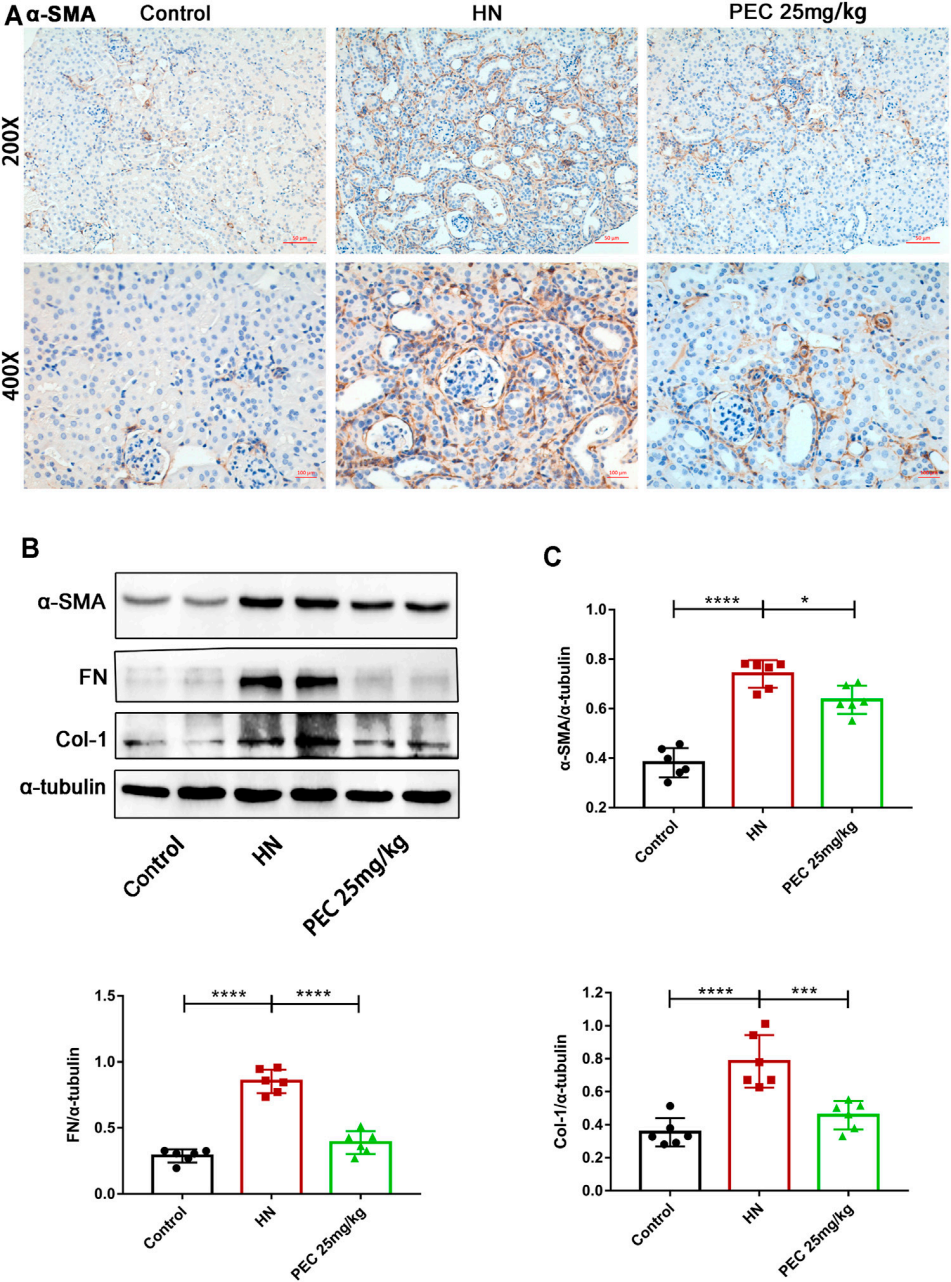
Effects of PEC on kidney fibrotic expression in HN mice. **(A)** Photomicrographs of α-SMA immunostaining in kidneys of mice (×200 and ×400). **(B,C)** The α-SMA, FN, and Col I protein levels normalized by α-tubulin. All data are represented as the mean ± SD. *p < 0.05, ***p < 0.001, ****p < 0.0001.

Consist with what transcriptome analysis found, the results from our western blot analysis showed that HN-induced kidney expression of apoptotic indicators was alleviated by PEC treatment (Figures 3A,B) (*p* < 0.05). In addition, the expression of proinflammatory cytokines (IL-6, TNF-α, MCP-1) was significantly increased in kidneys of HN mice and further decreased by PEC treatment (Figure 3C) (*p* < 0.05). Moreover, Masson’s staining (blue) revealed a remarkable increase of renal interstitial fibrosis in HN mice, which was ameliorated by PEC (Figure 4) (*p* < 0.05). Accordingly, the elevated accumulation of fibrotic markers of α-SMA, Col I, and FN was observed in kidneys of HN mice, and PEC significantly reduced the accumulation of these corresponding genes (Figure 5) (*p* < 0.05). The above results illustrated that PEC alleviated renal apoptosis, inflammation, and fibrosis in HN mice.

### Pectolinarigenin Downregulated the Expression of FABP4 in the Kidneys of Hyperuricemic Nephropathy Mice

**FIGURE 6:**
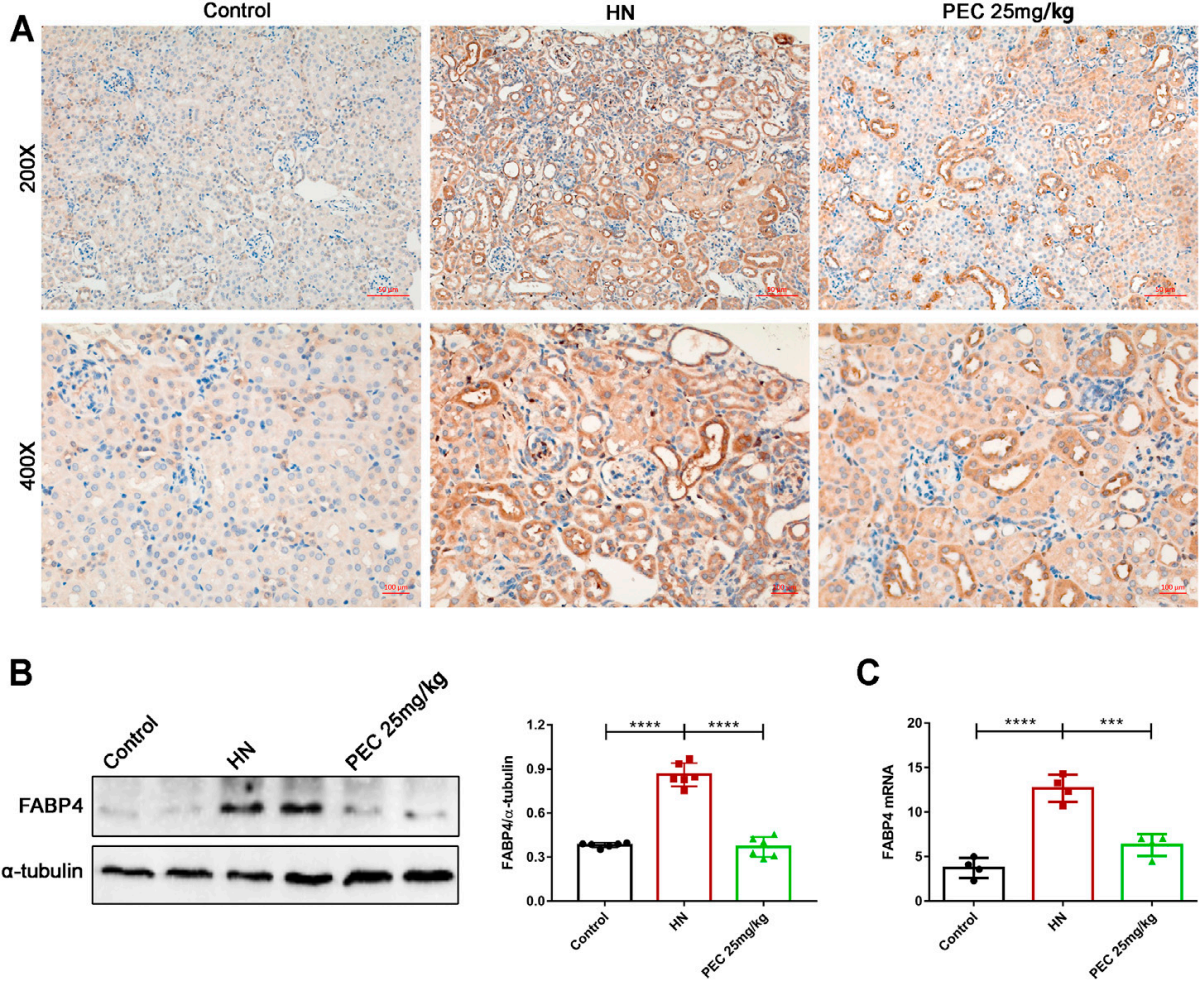
Effects of PEC on FABP4 expression in kidneys of HN mice. **(A)** Photomicrographs of α-SMA immunostaining in kidneys of mice (×200 and ×400). **(B)** The FABP4 protein level normalized by α-tubulin. **(C)** The mRNA expressions of FABP4 measured by real-time PCR analysis. All data are represented as the mean ± SD (*n* = 3). ***p < 0.001, ****p < 0.0001.

Our early study indicated that the lipid-binding chaperone FABP4 was increased in kidneys of HN mice and played crucial role in HUA-induced renal inflammation and fibrosis (Shi et al., 2020a). In line with our previous findings, the expression of FABP4 in kidneys of HN mice was significantly increased (*p* < 0.05). PEC treatment largely inhibited the expression of FABP4 both in the mRNA and protein level, further demonstrating the role of PEC in HUA-induced inflammation and fibrosis (Figure 6) (*p* < 0.05).

### Pectolinarigenin Suppressed the TGF-β/SMAD3 and JAK2/STAT3 Signaling Pathway in the Kidneys of Hyperuricemic Nephropathy Mice

**FIGURE 7:**
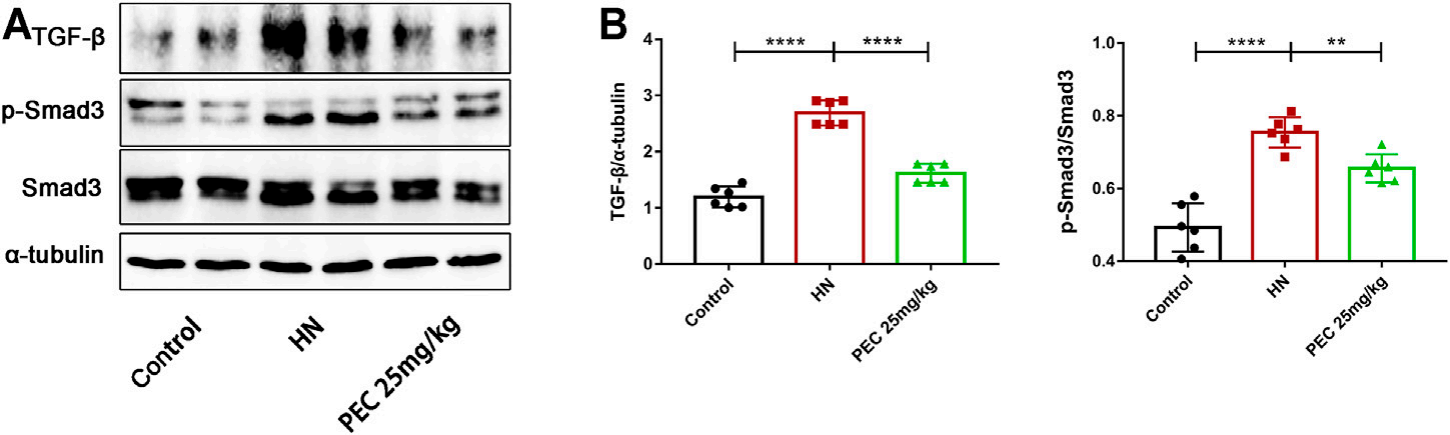
Effects of PEC on the activation of TGF-β/SMAD3 signaling in kidneys of HN mice. **(A,B)** The TGF-β and p-Smad3/Smad3 protein levels normalized by α-tubulin. All data are represented as the mean ± SD (*n* = 3). **p < 0.01, ****p < 0.0001.

As the most potent fibrogenic factor, TGF-β was considered to contribute to HUA-mediated renal fibrosis via the activation of Smad3 (Liu et al., 2015). To investigate the effect of PEC on the activation of TGF-β/Smad3 signaling in mice of HN, we measured the expression of TGF-β by western blot analysis. It was shown that TGF-β expression was significantly increased in kidneys of HN mice and decreased by PEC treatment (Figure 7) (*p* < 0.05). Meanwhile, kidney injury resulted in the phosphorylation of Smad3, which was remarkedly suppressed by PEC (Figure 7) (*p* < 0.05). Altogether, these results suggested that PEC could inhibit activation of TGF-β/Smad3 signaling pathway in the kidneys of HN mice.

**FIGURE 8:**
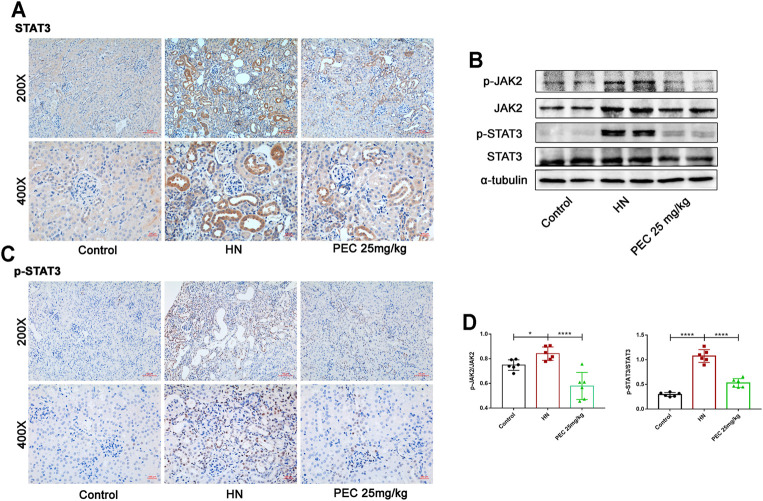
Effects of PEC on the activation of JAK2/STAT3 signaling in kidneys of HN mice. **(A,B)** Photomicrographs of STAT3 and p-STAT3 immunostaining in kidneys of mice (×200 and ×400). **(C,D)** The p-JAK2/JAK2 and p-STAT3/STAT3 protein levels normalized by α-tubulin. All data are represented as the mean ± SD (*n* = 3). *p < 0.05, * ****p < 0.0001.

STAT3 is a cytoplasmic transcription factor that could elicit diverse biological outcomes. Considerable studies have elucidated the role of STAT3 in mediating HUA-induced renal inflammation, apoptosis, and fibrosis (Shi et al., 2020b; Pan et al., 2021). To examine whether PEC could abrogate the activation of STAT3 in HN, the immunochemical staining and western blot analysis was employed to measure the expression of phosphorylated STAT3 (p-STAT3). As shown by Figure 8, the phosphorylation level of STAT3 was significantly increased in kidneys of HN mice, which was restored by PEC (Figure 8) (*p* < 0.05). Additionally, immunochemical staining showed that HN-induced p-STAT3 was mainly located in renal tubules (Figures 8A,B).

### Pectolinarigenin Inhibited Proinflammatory and Fibrotic Expression in Uric Acid-Stimulated TCMK-1 Cells

**FIGURE 9:**
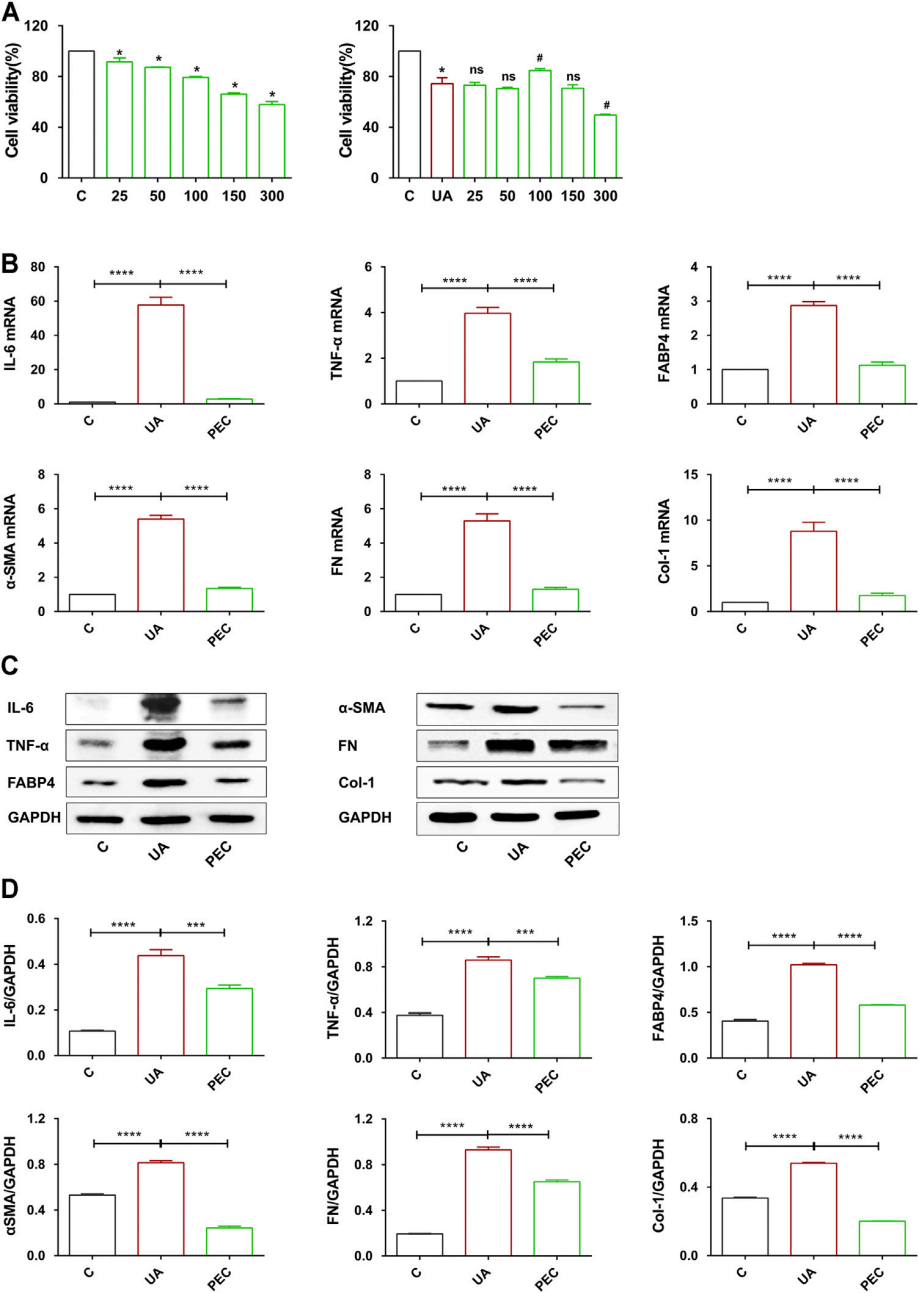
Effects of PEC on the proinflammatory and fibrotic expression in UA-treated TCMK-1 cells. **(A)** Cytotoxicity of PEC-treated TCMK-1cells with or without UA. **(B)** The mRNA expressions of IL-6, TNFα, FABP4, α-SMA, FN, and Col I measured by real-time PCR analysis. **(C,D)** The IL-6, TNFα, FABP4, α-SMA, FN, and Col I protein levels normalized by GAPDH. All data are represented as the mean ± SD (*n* = 3). *p < 0.05, ***p < 0.001, ****p < 0.0001, #*p* < 0.05 compared with UA-treated cells, ns means no significance.

To further investigate the role of PEC in HN, TCMK-1 cells were treated with soluble UA (800 μM) for 24 h. As shown in Figure 9A, PEC under 150 μM showed no cytotoxic effect for TCMK-cells and cells treated with PEC at 100 μM showed the highest cell viability. UA stimulation led to increased expression of IL-6, TNF-α, and FABP4 in TCMK-1 cells, and PEC (100 μM) significantly suppressed such expression (Figures 9B–D) (*p* < 0.05). Meanwhile, the fibrotic expression of α-SMA, FN, and Col I in UA-treated TCMK-1 cells was reduced by PEC (100 μM) (Figures 9B–D) (*p* < 0.05), thus confirming the anti-inflammatory and anti-fibrotic effects of PEC *in vitro*.

### Pectolinarigenin Hindered TGF-β/SMAD3 and JAK2/STAT3 Activation in Uric Acid-Induced TCMK-1 Cells

**FIGURE 10:**
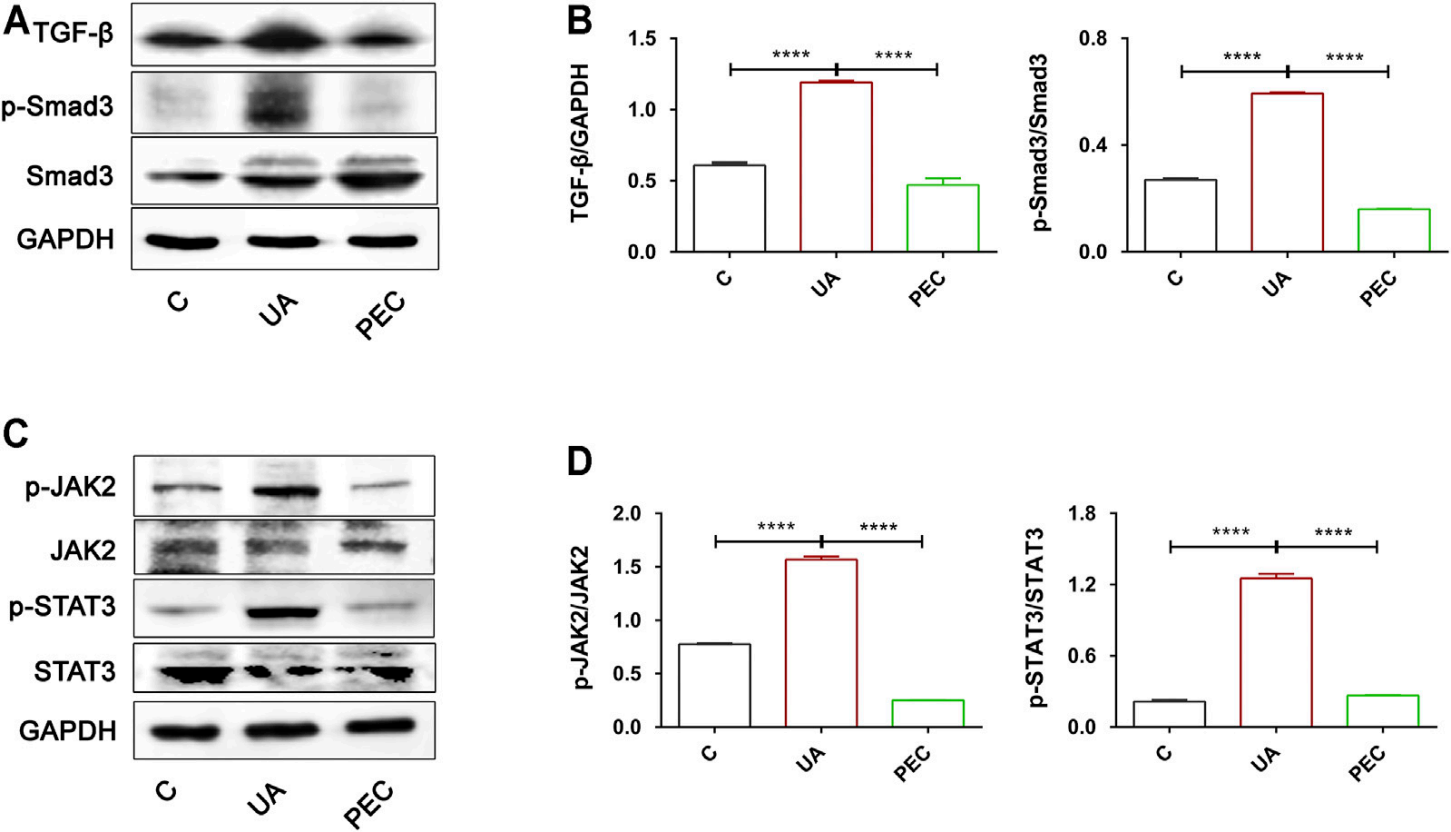
Effects of PEC on the activation of TGF-β/SMAD3 and JAK2/STAT3 signaling pathways in UA-stimulated TCMK-1 cells. **(A,B)** The TGF-β and p-Smad3/Smad3 protein levels normalized by GAPDH. **(C,D)** The p-IAK2/JAK2 and p-STAT3/STAT3 protein levels normalized by α-tubulin. All data are represented as the mean ± SD (*n* = 3). ****p < 0.0001.

After UA treatment, the expression of TGF-β and phosphorylated Smad3 were significantly increased in TCMK-1 cells, indicating that HUA could directly activate the TGF-β/Smad3 signaling pathway (Figures 10A,B) (*p* < 0.05). PEC (100 μM) successfully suppressed the expression of TGF-β and the phosphorylation of Smad3 indued by UA (Figures 10A,B) (*p* < 0.05). Similarly, UA stimulation resulted in the phosphorylation of JAK2 and STAT3, which was abrogated by PEC treatment (100 μM) (Figures 10C,D) (*p* < 0.05). Hence, consistent with our *in vivo* findings, PEC (100 μM) could inhibit the TGF-β/Smad3 and JAK2/STAT3 activation in UA-treated TCMK-1 cells.

## Discussion

Generally, UA is an antioxidant agent in a physiological medium (Dalbeth et al., 2021). Disturbance of the balance between UA production and excretion would lead to HUA that is considered as an independent risk factor for CKD progression (Johnson et al., 2013). Persistently high-serum UA levels was reported to trigger kidney inflammation and fibrosis that might contribute to HN (Lee et al., 2021). Current standard treatment for HUA is UA-lowering drugs represented by XO inhibitors and uricosuric agents, the nephroprotective effect of which remains controversy in CKD patients (Liu et al., 2021). Consequently, novel effective drugs for the prevention and treatment of HN need to be explored.

PEC is a natural flavonoid that showed therapeutic potential for inflammatory diseases, diabetes, and several types of cancers (Cheriet et al., 2020). Meanwhile, PEC could alleviate renal fibrosis in mice undergoing unilateral ureteral obstruction (UUO) (Li et al., 2021). However, the effects and underlying mechanism of PEC against HN remains unclear. In the present study, we noticed that PEC improved both HUA and renal damage in adenine and potassium oxonate-treated mice, as evidenced by reduced serum levels of UA, blood urea nitrogen (BUN), and creatinine and attenuated renal pathological changes. Remarkably, it was noticed that PEC at a dose of 25 mg/kg was more efficient in alleviating above biochemical parameters than PEC at a dose of 50 mg/kg. This might be explained by side effects of increased dosage as cytotoxicity effects of PEC have been reported by early studies (Lee et al., 2018). Moreover, PEC attenuated HUA-induced apoptosis characterized by the imbalance of Bcl-2/Bax and increased expression of cleaved caspase 3, suggesting the nephroprotective effects of PEC in HN.

Accumulation of UA increased the levels of inflammatory cytokines to mediate kidney injury (Li et al., 2016; Xu et al., 2021). In line with this, the elevated expression of IL-6, TNF-α, and MCP-1 were noticed in kidneys of HN mice and UA-stimulated TCMK-1cells, which was inhibited by PEC. The lipid transporter FABP4 is a potential mediator of inflammatory responses that has been suggested to play a crucial role in mediating renal inflammation and fibrosis in HN (Hotamisligil and Bernlohr, 2015; Shi et al., 2020a). Our results showed that PEC suppressed HUA-induced FABP4 expression, further illustrating its anti-inflammatory effects of PEC in HN.

Kidney fibrosis, the ultimate pathological outcome of HN, is characterized by the expression of mesenchymal cell products such as α-SMA, FN, and Col I (Lee et al., 2021). The TGF-β/Smad3 signaling pathway plays a critical role in mediating profibrotic response of renal epithelial cells and activating renal fibroblasts (Liu et al., 2015). TGF-β interacts with its receptors to phosphorylate Smad2/3 and subsequently regulates the transcription of profibrotic genes (Zhang et al., 2018). It was observed that HUA activated the TGF-β/Smad3 signaling pathway in HN mice (Balakumar et al., 2020; Shi et al., 2020b). In this study, we noticed that PEC successfully diminished HUA-induced TGF-β expression and Smad3 phosphorylation, which is in agreement with our previous finding that PEC blocked TGFβ1-induced SMAD3 phosphorylation in fibroblast (Li et al., 2021). Meanwhile, PEC significantly reduced the expression of α-SMA, FN, and Col I induced by HUA, suggesting that PEC suppressed the TGFβ1/Smad3 signaling pathway to alleviate kidney fibrosis in HN mice.

Considerable studies have implicated that activation of STAT3 via the IL-6/JAK2 cascade mediated inflammation and fibrosis in HN (Ren et al., 2021; Wu et al., 2021). Pharmacological inhibition of STAT3 was reported to attenuate kidney injury, slow down fibrosis, and suppress multiple proinflammatory cytokine production in kidneys of HN mice (Pan et al., 2021). Studies have identified PEC as a STAT3 inhibitor to suppress tumor growth and metastasis (Zhang et al., 2016; Gan et al., 2019; Li et al., 2019). Our previous study also indicated that PEC inhibited the activation of STAT3 in kidneys of UUO mice (Li et al., 2021). In the current study, treatment with PEC suppressed the phosphorylation of STAT3 signaling in kidneys of HN mice and UA-induced TCMK-1 cells, which might be the mechanism by which PEC ameliorated kidney inflammation and fibrosis in HN.

In summary, anti-hyperuricemic and nephroprotective effects of PEC were firstly demonstrated in adenine and potassium oxonate-induced HN mice and UA-treated TCMK-1 cells. Our results suggested that PEC attenuated kidney inflammation and fibrosis induced by HUA. Mechanically, we found that the nephroprotective effects of PEC were associated with the inhibition of the Smad3 and STAT3 signaling pathways. Taken together, PEC may be a candidate drug for the treatment of hyperuricemic nephropathy.

## Data Availability

The data presented in the study are accessible in the GEO repository, accession number GSE190205. The study can also be seen at: https://www.ncbi.nlm.nih.gov/geo/query/acc.cgi?acc=GSE190205
