# Supplementary material for: Natural Flavonoid Pectolinarigenin Alleviated Hyperuricemic Nephropathy via Suppressing TGFβ/SMAD3 and JAK2/STAT3 Signaling Pathways
Source: Front Pharmacol. 2022 Jan 27;12:792139. doi: 10.3389/fphar.2021.792139 (PMC8829971; doi:10.3389/fphar.2021.792139)
Supplement: Supplementary file 1 [file Table1.DOCX]

Supplementary Material

# Supplementary Table

Supplementary Table 1. Sequences of the primers for real-time PCR

| Mouse Gene | Sequence |
| --- | --- |
| *F-FABP4* | GGGGCCAGGCTTCTATTCC |
| *R-FABP4* | GGAGCTGGGTTAGGTATGGG |
| *F-IL-6* | ACAACCACGGCCTTCCCTACTT |
| *R-IL-6* | CACGATTTCCCAGAGAACATGTG |
| *F-TNF-α* | ACCCTCACACTCAGATCATCTTC |
| *F-TNF-α* | TGGTGGTTTGCTACGACGT |
| *F-MCP-1* | CATCCACGTGTTGGCTCA |
| *R-MCP-1* | GATCATCTTGCTGGTGAATGAGT |
| *F-Col1a1* | TGCCGCGACCTCAAGATGTG |
| *R- Col1a1* | CACAAGGGTGCTGTAGGTGA |
| *F-Fn* | GCAAACCTATAGCTGAGAAGTG |
| *R-Fn* | CAAGTACAGTCCACCATCATC |
| *F-α-SMA* | TCTCAAACATAATCTGGGTCA |
| *R-α-SMA* | CAGGGAGTAATGGTTGGAAT |
| *F-GAPDH* | GTATGACTCCACTCACGGCAAA |
| *R-GAPDH* | GGTCTCGCTCCTGGAAGATG |
